# Concrete Crack Detection and Monitoring Using a Capacitive Dense Sensor Array

**DOI:** 10.3390/s19081843

**Published:** 2019-04-18

**Authors:** Jin Yan, Austin Downey, Alessandro Cancelli, Simon Laflamme, An Chen, Jian Li, Filippo Ubertini

**Affiliations:** 1 Department of Civil, Construction and Environmental Engineering, Iowa State University, 813 Bissell Road, Ames, IA 50011, USA; acancell@iastate.edu (A.C.); laflamme@iastate.edu (S.L.); achen@iastate.edu (A.C.); 2 Department of Mechanical Engineering, University of South Carolina, 300 Main St, Columbia, SC 29208, USA; austindowney@sc.edu; 3 Department of Civil, Environmental and Architectural Engineering, University of Kansas, Lawrence, KS 66045, USA; jianli@ku.edu; 4 Department of Civil and Environmental Engineering, University of Perugia, 06125 Perugia, Italy; filippo.ubertini@unipg.it

**Keywords:** crack, strain, distributed dense sensor network, structural health monitoring

## Abstract

Cracks in concrete structures can be indicators of important damage and may significantly affect durability. Their timely identification can be used to ensure structural safety and guide on-time maintenance operations. Structural health monitoring solutions, such as strain gauges and fiber optics systems, have been proposed for the automatic monitoring of such cracks. However, these solutions become economically difficult to deploy when the surface under investigation is very large. This paper proposes to leverage a novel sensing skin for monitoring cracks in concrete structures. This sensing skin is constituted of a flexible electronic termed soft elastomeric capacitor, which detects a change in strain through changes in measured capacitance. The SEC is a low-cost, durable, and robust sensing technology that has previously been studied for the monitoring of fatigue cracks in steel components. In this study, the sensing skin is introduced and preliminary validation results on a small-scale reinforced concrete beam are presented. The technology is verified on a full-scale post-tensioned concrete beam. Results show that the sensing skin is capable of detecting, localizing, and quantifying cracks that formed in both the reinforced and post-tensioned concrete specimens.

## 1. Introduction

Cracks that manifest in concrete structures can be caused by a combination of poor construction practices, deleterious chemical reactions such as corrosion and alkali-aggregate reactions, construction overloads, cyclic freezing and thawing damage [1]. Cracks may represent the full extent of the damage or may point to problems of a larger scale. Their gravity depends on the type of structural system and the nature of cracking. If located at critical locations and of significant sizes, these cracks will decrease the capacity of the component and affect the durability and safety of the structure. A survey of cracks generally aids practitioners in evaluating and managing maintenance actions for a given structural system by providing information on the affected area, severity of the cracks, and their possible effect on structural integrity.

Various evaluation techniques can be leveraged during an inspection to determine the location and extent of cracking, and to evaluate the general condition of the concrete. These methods include visual inspections and nondestructive evaluation techniques such as impact-echo [2], ultrasonic [3], acoustic emission [4], and ground penetrating radar [5] methods. The advantage of leveraging nondestructive evaluation techniques during inspections is in their quantitative nature that can validate the subjective judgment of an inspector, but they yet require highly trained agents and expensive equipment. It must also be noted that inspections are typically conducted at fixed intervals, and it follows that they do not guarantee that critical damage will be detected timely.

A solution is the implementation of automated monitoring solutions, also known as structural health monitoring (SHM). Conventional SHM approaches to crack monitoring include resistive strain gauges [6], vibrating wires [7], and linear variable differential transformers [8]. While each technology has demonstrated success in certain conditions, they are limited by relatively small gauge lengths that impede their practicality for the monitoring of large-scale surfaces. Recently, electro-mechanical impedance (EMI) techniques using piezoelectric transducers (PZT) have been studied for real-time crack monitoring and early-damage detection [9]. These sensors exhibit a high sensitivity to crack growth. However, the disadvantage of PZTs is that they have low interface compatibility and poor durability when used for monitoring concrete structures [10]. Fiber optic sensors (FOS) that can be multiplexed for a long distance and immune to electromagnetic interference have gained popularity since the 1990s [11] to map cracks over large areas for both surface [12] and embedded [13] applications. Nevertheless, FOS technologies are still expensive to deploy, can be brittle, are challenging to bond onto surfaces, and embedment is limited to new retrofits and constructions.

Novel surface strain sensing technologies, or sensing skins, with excellent durability, sensitivity, and cost-effectiveness for geometrically large systems, have gained popularity in the research community as an organic step beyond FOS. These include strain sensing sheets based on large area electronics and integrated circuits [14,15,16], electrical impedance tomography (EIT) [17,18], and multifunctional materials [19,20]. The authors have previously proposed sensing skin technology based on soft elastomeric capacitors (SECs) that act as large-area strain gauges. The SECs offer unique advantages for crack detection and monitoring over traditional sensing technologies due to their low-cost [21], high durability to environmental conditions [22], and mechanical robustness [23]. Previous investigations have experimentally evaluated the feasibility of using SECs for fatigue crack localization and quantification in steel bridges [24].

This study aims at extending previous research efforts on the SECs to the monitoring of cracks in concrete. In particular, the performance of SECs at localizing and assessing flexural crack development in concrete infrastructures through strain measurements is evaluated on a small-scale and a full-scale concrete beam using a network of strip-shaped SECs. The remainder of the paper is organized as follows. Section 2 introduces the sensing principle of SECs and presents a verification of the sensing principle on a small reinforced concrete beam. Section 3 presents and discusses experimental results conducted on a full-scale post-tensioned concrete beam to validate the performance of the technology. Section 4 concludes the paper.

## 2. Soft Elastomeric Capacitor Technology

This section provides a background on the SEC technology, including its fabrication process and electromechanical model, and presents validation results on a small-scale concrete beam.

### 2.1. Sensor Fabrication

**Figure 1:**
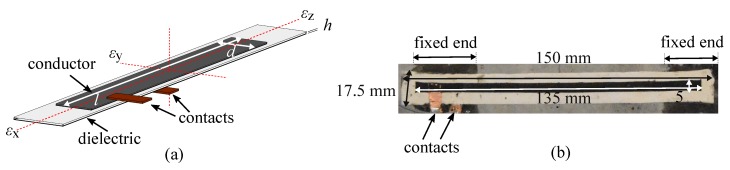
(**a**) Schematic representation of an SEC; (**b**) picture of an SEC with active sensing area measuring 135 mm × 5 mm.

The SEC is a low-cost, robust, and highly scalable thin-film strain sensor that consists of a flexible parallel plate capacitor. A given change in a monitored surface’s geometry (i.e., strain) is transduced into a measurable change in the SEC’s capacitance. An SEC is presented in Figure 1 with its key components annotated. The SEC is constituted from a styrene--ethylene/butylene--styrene (SEBS) block copolymer arranged in three layers. The inner layer (dielectric) is filled with titania to increase both its durability and permittivity, while the outer layers (conductors) are filled with carbon black to provide conductivity. The carbon black-filled outer layers also provide enhanced UV light protection, therefore enhancing the sensor’s environmental durability [22]. The fabrication process of the SEC is covered in more detail in [23]. An electromechanical model that relates a change in the monitored structure geometry (i.e., strain) to a change in the sensor’s capacitance (*C*) can be derived from the parallel plate capacitor equation:

(1) $$C=e_{0}e_{r}\frac{A}{h}$$

where $e_{0}$ = 8.854 pF/m is the vacuum permittivity, $e_{r}$ is the polymer’s relative permittivity, A=d·l is the sensor area of width *d* and length *l* (as annotated in Figure 1a), and *h* is the thickness of the dielectric.

Equation (1) can be specialized for the sensor configuration of interest to this paper, where the sensor is glued at each end and free-standing in the middle, as shown in Figure 1b, undergoing uniaxial strain ($\varepsilon =\varepsilon_{x}$):

(2) $$\varepsilon =\lambda \frac{∆C}{C_{0}}=\frac{∆l}{l_{0}}$$

where $l_{0}$ is the unstrained length of the SEC, $C_{0}$ the initial unstrained capacitance, ∆C the incremental change in capacitance and λ the gauge factor. In the sensor configuration of interest, the gauge factor is a function of the sensor geometry. The sensor’s general overall dimensions used in this study are 150 mm × 17.5 mm, with the active sensing area measuring 135 mm × 5 mm (Figure 1b). The next subsection characterizes such SEC’s response to determine the gauge factor λ.

### 2.2. Sensor Response Characterization

**Figure 2:**
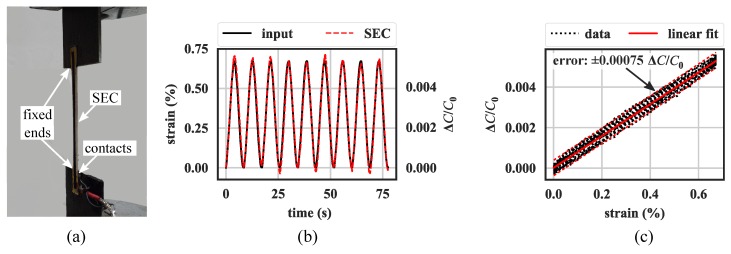
(**a**) Experimental gauge factor characterization test setup; (**b**) capacitance time history response subject to cyclic strain input; and (**c**) sensitivity and linearity of the sensor.

The electromechanical response of an end-bonded SEC (as shown in Figure 1b) was investigated by applying an axial 0.12 Hz cyclic excitation on a free-standing specimen using a servo-hydraulic testing machine, as shown in Figure 2a. During the test, the SEC’s capacitance was recorded at 24 Hz using a custom-built data acquisition device (DAQ), and the displacement response was recorded at 600 Hz from the dynamic testing machine. Figure 2 presents the results of the electromechanical test, comparing the measured strain (black line in Figure 2b) and the corresponding change in capacitance measured by the SEC. Figure 2c reports the change in capacitance as a function of the change in strain. As shown in Figure 2c, the strain and measured change in capacitance have a linear relationship that when fitted with linear least squares regression can be used to obtain the gauge factor λ=0.78 over the tested range 0–0.7% strain. Figure 2c shows the capacitance error bound (±0.00075 $∆C/C_{0}$), equivalent to a resolution of 9.6 με.

Note that the capacitance is expected vary linearly with temperature and humidity [25]. However, the sensor’s response with respect to strain (i.e., gauge factor) will remain constant. A thorough study of environmental effects in terms of sensor weatherability and long-term signal stability could be found in Reference [22].

### 2.3. Small-Scale Prototyping Test

**Figure 3:**
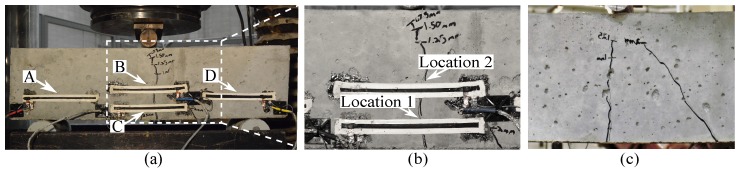
(**a**) Crack patterns on the small-scale reinforced concrete beam; (**b**) an enlarged view of the cracks on the front side of the specimen; and (**c**) an enlarged view of the cracks on the back side of the specimen showing a shear crack.

To investigate the feasibility of the proposed approach, an experimental campaign was conducted on two small-scale reinforced concrete beams and results published in a conference proceeding [26]. This subsection presents typical results to validate the sensor’s capabilities. The testing specimens were subjected to a three-point bending test to study the detectability of bending cracks using an SEC array. The dimension of the small-scale reinforced concrete beam was 61 cm × 15 cm × 15 cm. The specimen is shown in Figure 3a and was equipped with an array of four sensors identified as SEC A, B, C and D. SECs B and C were both placed at midspan but at different heights to study additional crack assessment capability. The remaining two SECs were placed symmetrically around the midspan.

**Figure 4:**
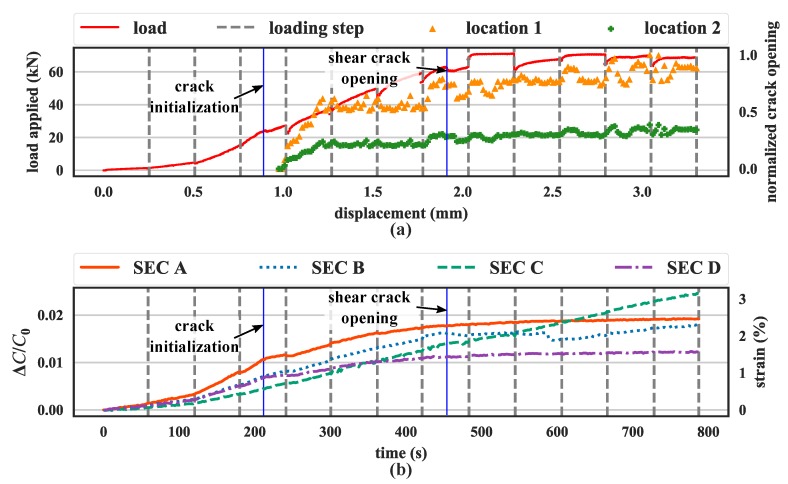
Time series results from the small-scale reinforced concrete beam: (**a**) load-displacement curve and normalized crack opening widths histories; and (**b**) relative capacitance and computed strain histories for all four SECs.

Figure 3b is a picture of the final crack pattern, and Figure 4 plots a time history of data. Note that the magnitude of strain in Figure 4 is very high, because it represents the strain experienced by the sensor spanning the crack opening. The gray dashed lines in Figure 4 show when the machine paused and resumed to produce incremental loads. The testing machine did not maintain a stable load while paused. This was confirmed through the analysis of the load-displacement curve of the specimen, along with the normalized crack width amplitude obtained by averaging the crack width at the top and bottom edges of the SEC (Figure 3a). For low levels of displacement, a single crack formed initially at the bottom-center of the beam. This single crack formation was confirmed through the slight drop in the load-displacement curve (the blue solid line between the third and fourth gray dashed line from the left in Figure 4a), along with the opening of the crack at mid-span (termed Location 1, illustrated with green dots, and Location 2 on top of Location 1 illustrated with orange triangles in Figure 4a). The flexural crack propagated to the compression zone, with a decrease of stiffness, up to a 2 mm displacement. There is a loss of capacity around 1.9 mm (second blue solid line in Figure 4a) which was produced by a shear crack opening on the back side of the concrete specimen (Figure 3c). From then up to 3.6 mm, the load remained constant. This behavior was almost completely captured by the SEC network installed on the specimen. As shown in Figure 4b, SECs A, B, and D have a decrease in slope that matches the one associated with the formation of the first flexural crack. At the initialization of the backside shear crack, SEC B was able to capture this behavior as a drop in its capacitance followed with an unstable capacitance growth (the second blue solid line from the left in Figure 4b), which may be associated with stress redistribution. One can also observe that SEC C, mounted at the bottom of the tension zone, should have the highest strain value/relative capacitance change, but it has the lowest change in relative capacitance at the initial stage. This could be attributed to strain transfer at the sensor interface caused by the installation procedure, which cannot be quantified directly, but does not significantly affect its capability to measure crack [27].

## 3. Verification on Full-Scale Post-Tensioned Concrete Beam

This section presents and discusses results on an experimental verification conducted on a full-scale post-tensioned concrete beam.

### 3.1. Experimental Structural System

The full-scale post-tensioned concrete beam of interest is part of a structural system consisting of two parallel full-scale beams connected by a deck. The structural system was tested within the scope of an unrelated research project. The installation of a network of SECs was allowed onto the side surface of one of the beams. During testing, the structural system was subjected to several damage scenarios, which included the removal of the post-tension in one of the girders and a large number of damage states ranging from incipient to severe damages and until failure.

**Figure 5:**
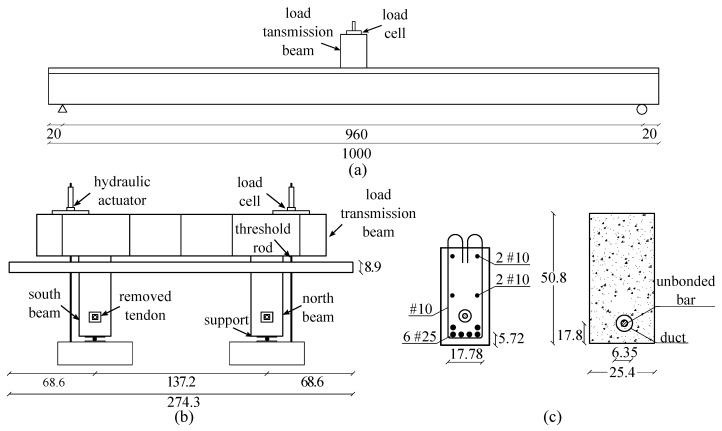
Detailing of the tested specimen: (**a**) elevation view; (**b**) typical cross-section; and (**c**) reinforcement distribution and cross-section design of a beam (dimensions are in cm).

Each beam has a rectangular cross-section of 254 mm × 508 mm, a length of 10 m, and a clear span of 9.6 m, as illustrated in Figure 5. The beam dimensions were designed to limit the first natural frequency of the full system below 12 Hz, which is a typical value for short-span bridges. A 391 kN post-tension force was applied to both beams before the casting of the connecting deck to avoid early cracking under self-weight. A circular plastic duct of 63.5 mm was installed in the cross section with its center located at 178 mm from the bottom face of the beam to accommodate a single post-tension bar of 25.4 mm diameter. The sections were reinforced using 6 reinforcement bars with a diameter of 25.4 mm, with their positions indicated in Figure 5 to provide high ductility of the girders. The deck connecting the two girders had a width of 3 m, thickness of 9 cm, and length equal to that of the beams (10 m). The deck was designed to ensure that cracks would only form on the girders, except around ultimate strength. The deck was reinforced using two layers of reinforcement bars of 12.7 mm diameter under both the bottom and top surfaces. The beams were positioned at 1.4 m from each other, leaving an overhang of 70 cm on each side as shown in Figure 5b. The structural system was loaded using hydraulic actuators (Figure 5b) installed over a beam transmitting the actuator force to the beams’ centerline. The girders and the deck were casted in Iowa State University’s Structures Laboratory using a self-compacting concrete with specified compressive strength of 41 MPa and 28 MPa, respectively. During tests, the measured compressive strength for the girders and deck were 48 MPa and 28 MPa, respectively.

**Figure 6:**
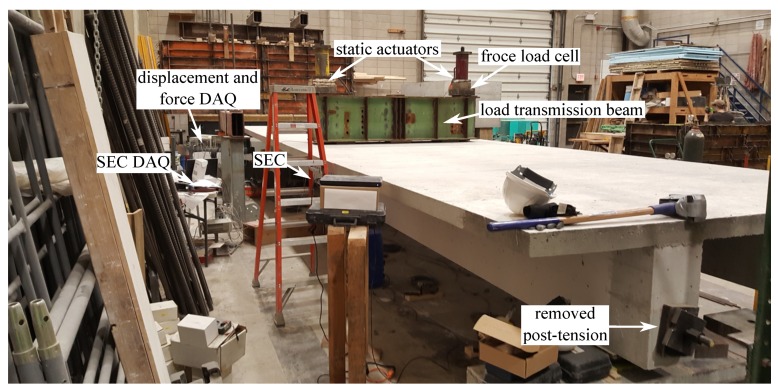
Picture showing the experimental setup of the large-scale post-tension beam test setup.

**Figure 7:**
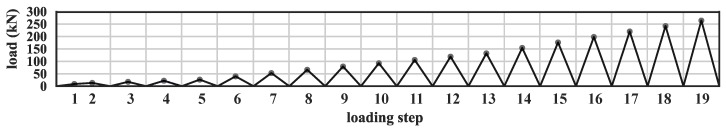
Loading-unloading sequence for the load test.

The experiment was conducted in two sequential phases. In the first phase, the prestress was released at one side of the beam (Figure 6) to provide differential damage. In the second phase, a load was applied using the pair of hydraulic force actuators installed on top of load transmission beam (Figure 6), and a load cell was used to constantly monitor the applied load. Loading and unloading sequences were designed to generate damage to the beams in increasing severity. Visual observations were conducted during the unloading phases. Figure 7 is a plot of the loading history.

#### Dense Sensor Network Instrumentation Strategy

**Figure 8:**
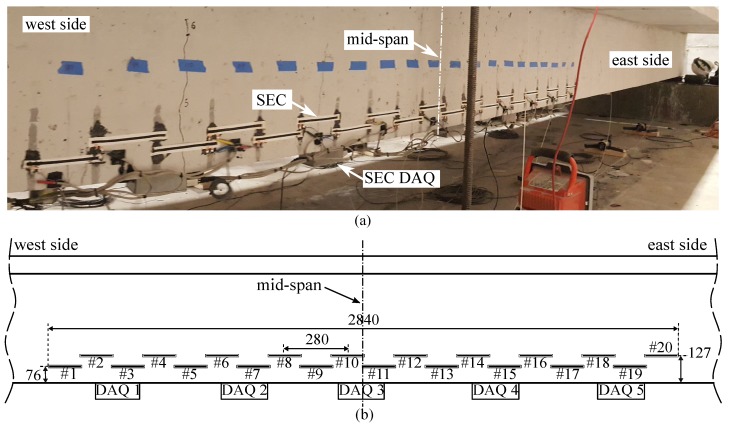
Sensor instrumentation around the midspan of the beam: (**a**) picture; and (**b**) schematic of sensor locations, partial beam elevation (dimensions are in mm).

In this investigation, an SEC array was instrumented on a post-tensioned concrete beam which post-tension was released. A total of 20 SECs were placed in order to cover 2.84 m of the beam centered around the beam’s midspan to study the evolution of spatial crack distribution. Based on the validation results from the small-scale beams (Section 2), the array was designed in an overlapping staggered pattern, as shown in Figure 8, to improve the probability of detection of all cracks. SECs were placed at 127 mm and 76 mm along the vertical direction from the bottom surface of the beam with a 280 mm spacing. Each SEC was pre-stretched and affixed onto the concrete substructure at two ends using a thin layer of an off-the-shelf epoxy (JB Kwik). It follows that the effective strain-sensitive portion of each sensor was narrowed to the unglued section. The numbering scheme of the SECs is shown in Figure 8. Capacitance data collected at 24 Hz using a customized DAQ (Figure 8) driven by a LabVIEW code.

### 3.2. Results and Discussion

**Figure 9:**
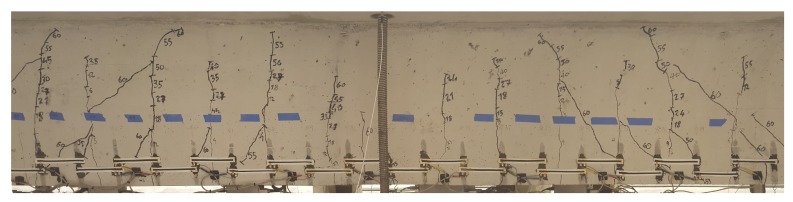
Picture of crack pattern before reaching ultimate strength after loading step 19.

Figure 9 is a picture showing the crack pattern at the end of loading step 19, with visible cracks traced using a black marker. The beam experienced uniformly distributed transverse and shear cracks at both two ends. The area under the loading point experienced shorter cracks, which is as expected, given the pressure from the flange restraining the crack growth. Cracks were observed in the loading zone under SEC #9, and #12 after loading step 2, under SECs #6, #7, #15, #16, #18, and #20 after loading step 3, and under SEC #1, #3 after loading step 4. Subsequent loading steps 5 to 17 induced a uniform formation and growth of flexural cracks along the span of the girder. Rapid formation of shear cracks crossing different SECs at both ends, from SEC #1 to #7 and from SEC #16 to #20, occurred after loading step 18 and 19.

**Figure 10:**
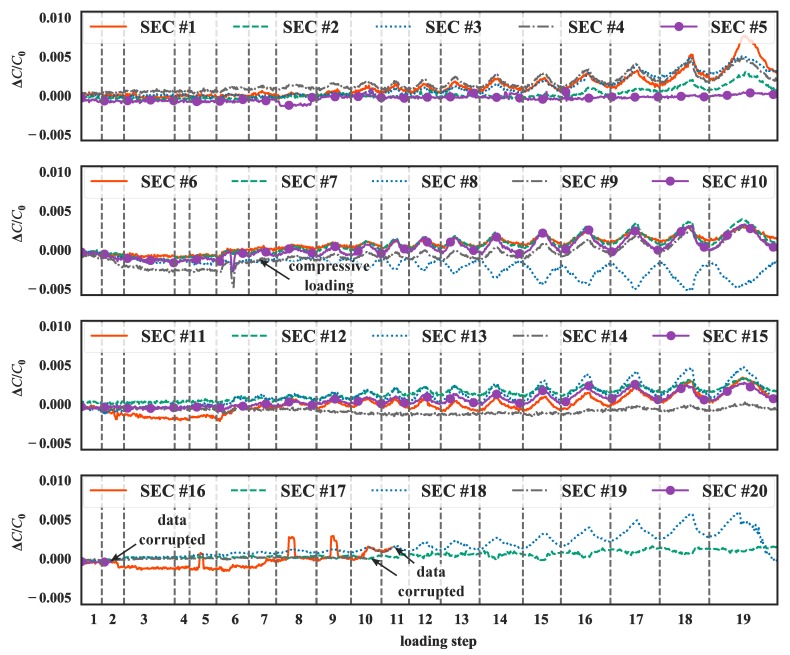
Time histories of strain measurements for all SECs.

Figure 10 plots the time evolution of the relative changes in capacitance $∆C/C_{0}$ during the loading test, where the gray dashed lines indicate when the machine paused after each incremental loading step was produced. Negative $∆C/C_{0}$ values indicate compression, while conversely a positive value indicates tension. Results are presented after the replacement of outliers with averaged values and the application of a low-pass Butterworth filter with a cutoff frequency of 2 Hz. SECs #16, #19 and #20 experienced corrupted data after a crack formed under the epoxy adhering the sensor to the beam.

SEC #8 exhibited a negative capacitance change and an inverse loading-unloading shape. This disagreement between the capacitance change and loading was caused by localized compressive strains induced by the splitting of the specimen along the flexural crack. This splitting in the specimen caused the right-hand-side portion of the crack under SEC #7 to move towards the right, thus resulting in compressive loading of the concrete under SEC #8. This behavior was confirmed when the flexural crack opened under SEC #9 at loading step 8, at which point the amplitude of the maximum cracks began to increase significantly. A similar, yet smaller in magnitude, compression behavior was observed from SEC #17 that was located between two cracks. Under loading step 5, the relative capacitance did not change significantly and the compressive effect induced from the flange at the center of the beam was captured by most SECs. The loading-unloading patterns in relative capacitance changes were observable in SEC readings after a crack formed under that particular SEC.

**Figure 11:**
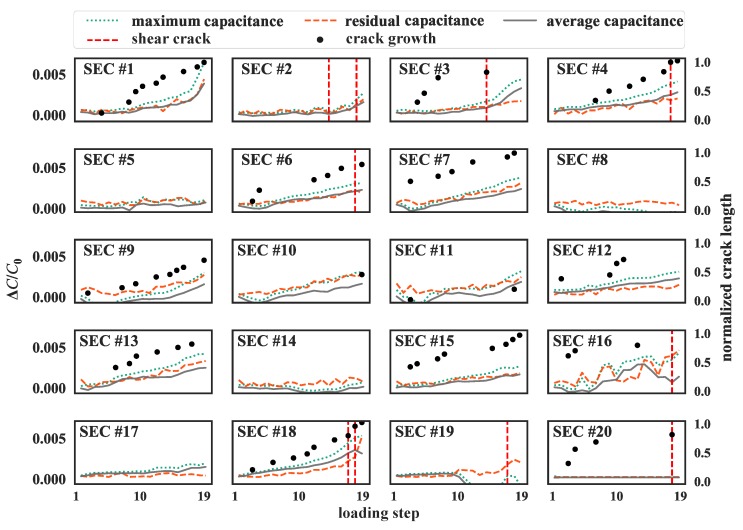
Comparison of maximum, residual, and average relative change in capacitance measured by the SECs and normalized crack growth length under each loading step (loading step 1 to 19).

In order to evaluate the performance of the SECs at quantifying crack openings, the relationship between crack growth and relative capacitance change was investigated. Three features were extracted from time series data to associate with crack length: (1) maximum relative change in capacitance, taken as the peak-to-peak amplitude in signal for each load step; (2) residual relative change in capacitance, taken as the difference between the maximum reading during a given load step and the capacitance left after unloading; and (3) average relative change in capacitance, taken as the average change in capacitance over a loading step. Figure 11 plots the maximum relative change in capacitance (green dotted line), residual relative change in capacitance (orange dashed line), average relative change in capacitance (gray line) against crack growth (black dots) from each loading step (L1 to L19) and SEC. The red dashed lines indicate the visible shear cracks initiation underneath the corresponding SECs. The crack length was measured as a straight line between the extremities of a crack, and normalized by the height of the web. An agreement is observed between the three features and crack growth.

**Figure 12:**
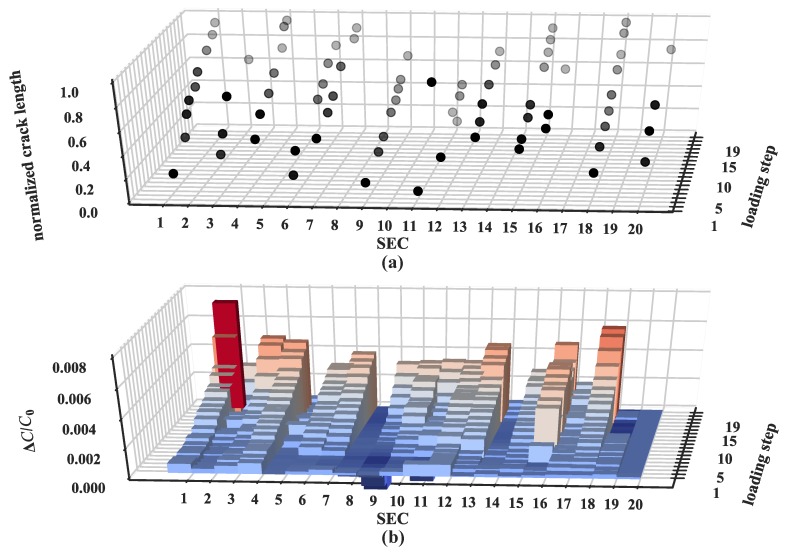
(**a**) Normalized crack length as a function of SEC location and loading step; (**b**) maximum relative change in capacitance change as a function of SEC location and loading step.

Figure 12 illustrates a compilation of the normalized crack lengths and the feature “maximum relative change in capacitance” extracted from each loading step for all the SECs. It can be observed that the feature is generally consistent with the location of a crack and its normalized length. At small strain (low loadings), this relationship is harder to distinguish. A significant change in the feature is associated with a rapid growth of the shear crack and final fracture, shown as tall red bars at the two corners of Figure 12b (SEC #1 and #18). Both small and negative changes in the feature indicate that no crack formed.

## 4. Conclusions

This paper presented the study of a novel sensing skin for the detection, localization, and quantification of cracks in concrete. The sensing skin, constituted from an array of soft elastomeric capacitors (SECs), is an inexpensive, durable, and robust sensing solution that can be leveraged to measure strain over large surfaces. The strain measurement values are collected in the form of discrete point values among the network. The spatio–temporal comparison of strains enables the detection, localization, and quantification of cracks.

The sensing skin was first introduced and validation results preliminarily conducted on small-scale reinforced concrete beams were presented. Initial characterization of a free-standing SEC led to a gauge factor that was used to map electrical signal to strain and thus, cracks. Time series measurements from the SECs and visual observations from crack growth were in agreement. The sensing capability was further studied by deploying a sensor network of 20 SECs onto the surface of a full-scale post-tensioned concrete beam. A bending test with a loading and unloading sequence was conducted until structural failure, and data from the SECs and visual observation of cracks collected. Results demonstrated that data collected from the distributed SEC network correlated with crack-induced damage. The extraction of time series features, among which the maximum relative change in capacitance at each loading step, showed good agreement with the observed normalized crack length.

Overall, the SEC network showed capable of detecting, localizing, and quantifying cracks in concrete. The application of dense networks of SECs could provide a cost-effective monitoring solution for real-time, long-term crack monitoring on civil structures. Future research will include the influence of the gauge length on the accuracy of SEC’s final configuration, bonding sensitivity between the concrete and sensing materials, optimal dense sensor network design for multi-crack detection, and crack initialization characterization algorithms.
